# Genomic prediction of coronary heart disease

**DOI:** 10.1093/eurheartj/ehw450

**Published:** 2016-09-21

**Authors:** Gad Abraham, Aki S. Havulinna, Oneil G. Bhalala, Sean G. Byars, Alysha M. De Livera, Laxman Yetukuri, Emmi Tikkanen, Markus Perola, Heribert Schunkert, Eric J. Sijbrands, Aarno Palotie, Nilesh J. Samani, Veikko Salomaa, Samuli Ripatti, Michael Inouye

**Affiliations:** 1 Centre for Systems Genomics, School of BioSciences, The University of Melbourne, Parkville, Victoria 3010, Australia; 2 Department of Pathology, The University of Melbourne, Parkville, Victoria 3010, Australia; 3 National Institute for Health and Welfare, Helsinki FI-00271, Finland; 4 Centre for Epidemiology and Biostatistics, Melbourne School of Population and Global Health, The University of Melbourne, Parkville, Victoria 3010, Australia; 5 Institute for Molecular Medicine Finland (FIMM), University of Helsinki, Helsinki FI-00014, Finland; 6 Deutsches Herzzentrum München, and Technische Universität München, Munich 80636, Germany; 7 Deutsches Zentrum für Herz- und Kreislauferkrankungen (DZHK), Partner Site Munich Heart Alliance, Munich 81377, Germany; 8 Department of Internal Medicine, Erasmus Medical Center, Rotterdam, CA 3000, The Netherlands; 9 Analytic and Translational Genetics Unit, Department of Medicine, Massachusetts General Hospital, Boston, Massachusetts 02114, USA; 10 Program in Medical and Population Genetics, Broad Institute of Harvard and MIT, Cambridge, Massachusetts 02142, USA; 11 Department of Psychiatry, Psychiatric & Neurodevelopmental Genetics Unit, Massachusetts General Hospital, Boston, Massachusetts 02114, USA; 12 Department of Cardiovascular Sciences, University of Leicester, BHF Cardiovascular Research Centre, Glenfield Hospital, Groby Rd, Leicester, LE3 9QP, United Kingdom; 13 National Institute for Health Research Leicester Cardiovascular Biomedical Research Unit, Glenfield Hospital, Groby Road, Leicester, LE3 9QP, United Kingdom; 14 Wellcome Trust Sanger Institute, Wellcome Trust Genome Campus, Hinxton, Cambridge CB10 1SA, United Kingdom; 15 Department of Public Health, University of Helsinki, Helsinki FI-00014, Finland

**Keywords:** Genomic risk score, Coronary heart disease, Myocardial infarction, Framingham risk score, Primary prevention

## Abstract

**Aims:**

Genetics plays an important role in coronary heart disease (CHD) but the clinical utility of genomic risk scores (GRSs) relative to clinical risk scores, such as the Framingham Risk Score (FRS), is unclear. Our aim was to construct and externally validate a CHD GRS, in terms of lifetime CHD risk and relative to traditional clinical risk scores.

**Methods and results:**

We generated a GRS of 49 310 SNPs based on a CARDIoGRAMplusC4D Consortium meta-analysis of CHD, then independently tested it using five prospective population cohorts (three FINRISK cohorts, combined *n* = 12 676, 757 incident CHD events; two Framingham Heart Study cohorts (FHS), combined *n* = 3406, 587 incident CHD events). The GRS was associated with incident CHD (FINRISK HR = 1.74, 95% confidence interval (CI) 1.61–1.86 per S.D. of GRS; Framingham HR = 1.28, 95% CI 1.18–1.38), and was largely unchanged by adjustment for known risk factors, including family history. Integration of the GRS with the FRS or ACC/AHA13 scores improved the 10 years risk prediction (meta-analysis C-index: +1.5–1.6%, *P *<* *0.001), particularly for individuals ≥60 years old (meta-analysis C-index: +4.6–5.1%, *P *<* *0.001). Importantly, the GRS captured substantially different trajectories of absolute risk, with men in the top 20% of attaining 10% cumulative CHD risk 12–18 y earlier than those in the bottom 20%. High genomic risk was partially compensated for by low systolic blood pressure, low cholesterol level, and non-smoking.

**Conclusions:**

A GRS based on a large number of SNPs improves CHD risk prediction and encodes different trajectories of lifetime risk not captured by traditional clinical risk scores.

## Introduction

Early and accurate identification of individuals with increased risk of coronary heart disease (CHD) is critical for effective implementation of preventative lifestyle modifications and medical interventions, such as statin treatment.^1^^,^^2^ To this end, risk scores such as the Framingham Risk Score (FRS)^3^ and the American College of Cardiology/American Heart Association 2013 risk score (ACC/AHA13),^1^ based on clinical factors and lipid measurements, have been developed and are widely used. Although the scores can identify individuals at very high risk, a large proportion of individuals developing CHD during the next 10 years remain unidentified. In particular, they do not provide sufficient discrimination at a younger age when implementation of preventative measures is likely to provide the greatest long-term benefit. 

Genetic factors have long been recognized to make a substantial contribution to CHD risk.^4^ Although a positive family history is an independent risk factor for CHD, it may not completely and solely capture genetic risk. Recently, genome-wide association studies (GWAS) have identified 56 genetic loci associated with CHD at genome-wide significance.5–9 Studies of the predictive power of the top single nucleotide polymorphisms (SNPs) at some of these loci either individually or in combination have typically shown small improvements in CHD risk prediction,10–17 probably because together these variants only explain less than 20% of CHD heritability.^8^ As demonstrated recently for other traits such as height and BMI,^18^^,^^19^ the majority of unexplained heritability is likely hidden amongst the thousands of SNPs that did not reach genome-wide significance. Indeed, recent advances have shown that genomic prediction models that consider all available genetic variants can more efficiently stratify those at increased risk of complex disease.20–24 To leverage the maximum amount of information, we examined whether a genomic risk score (GRS) comprising a large number of SNPs, including those with less than genome-wide significance, could produce clinically relevant predictive power for CHD risk.

## Methods

**Figure 1:**
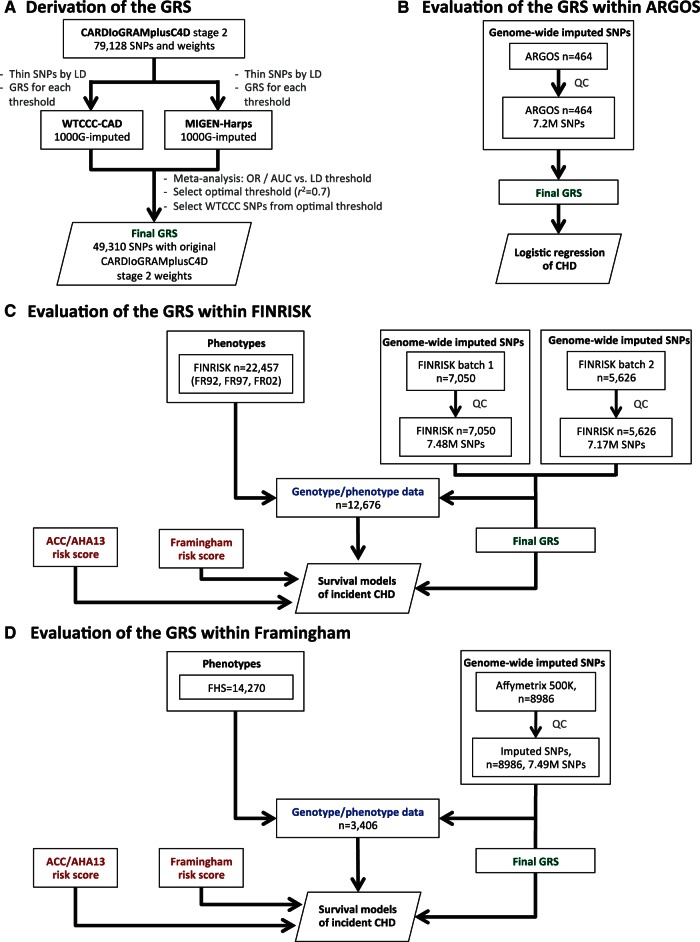
Study workflow. (*A*) The procedure for deriving the GRS of incident CHD. The analysis workflow for evaluating the GRS within (*B*) ARGOS, (*C*) FINRISK, and (*D*) FHS.

A summary of the key methods for the study is given here. The study design is given in *Figure 1*. Additional details are provided in the see Supplementary Data.

### Prospective study cohorts

We utilized two sets of prospective cohorts: (i) FINRISK, consisting of three prospective cohorts from Finland with 10–20 years of follow-up, from collections 1992, 1997, and 2002 (FR92, FR97, and FR02, respectively)^25^ and (ii) the Framingham Heart Study (FHS),26–28 with individuals of Western and Southern European ancestry taken from the Original and Offspring cohorts with 40–48 years of follow-up. In total, the FINRISK consisted of *n* = 12 676 individuals and the FHS of *n* = 3406 individuals, all of whom had the requisite data and were independent of the CARDIoGRAMplusC4D stage-2 meta-analysis utilized to generate the GRS (*Table 1*). The cohorts have been genome-wide SNP genotyped and further imputed to the 1000 Genomes reference panel (see Supplementary Data online, Supplementary Data). After genotype imputation and quality control, 69 044 autosomal SNPs of the 79 128 CARDIoGRAMplusC4D SNPs were available for subsequent analyses in the FINRISK, and 78 058 autosomal SNPs available in FHS.

**Table 1:** Characteristics of the FINRISK and FHS cohorts

Study	FINRISK				Framingham Heart Study		
Cohort	FR92 (*n*=3547)	FR97 (*n*=4761)	FR02 (*n*=4368)	Total FINRISK (*n*=12,676)	FHS Original (*n*=950)	FHS Offspring (*n*=2456)	Total FHS (*n*=3406)
Men	1578 (44%)	2316 (49%)	1919 (44%)	5813 (46%)	370 (39%)	1179 (48%)	1549 (45%)
Women	1969 (56%)	2445 (51%)	2449 (56%)	6863 (54%)	580 (61%)	1277 (52%)	1857 (55%)
Baseline age, years	43.59 (11.31)	46.68 (13.15)	47.12 (13.01)	45.97 (12.7)	53.7 (6.09)	40.66 (7.47)	44.3 (9.21)
Current smoker	1027 (29%)	1148 (24%)	1162 (27%)	3337 (26%)	422 (44%)	948 (39%)	1370 (40%)
Blood pressure, systolic, mm Hg	134.79 (19.13)	135.02 (19.62)	134.94 (20.24)	134.93 (19.7)	131.54 (19.35)	122.64 (15.98)	125.12 (17.45)
Cholesterol, total, mmol/L	5.6 (1.12)	5.54 (1.06)	5.62 (1.14)	5.58 (1.11)	6.14 (1.08)	5.21 (0.98)	5.47 (1.09)
Cholesterol, HDL, mmol/L	1.41 (0.35)	1.42 (0.35)	1.52 (0.43)	1.45 (0.38)	1.3 (0.37)	1.33 (0.39)	1.32 (0.39)
Prevalent type 2 diabetes	119 (3%)	299 (6%)	278 (6%)	696 (5%)	19 (2%)	39 (2%)	58 (2%)
Lipid lowering treatment	43 (1%)	117 (2%)	231 (5%)	391 (3%)	–	–	–
Anti-hypertensive treatment	302 (9%)	569 (12%)	582 (13%)	1453 (11%)	57 (6%)	75 (3%)	132 (4%)
Follow up, years	18.49 (3.77)	13.82 (2.88)	9.47 (1.51)	13.63 (4.53)	29.91 (11.32)	31.95 (8.44)	31.38 (9.38)
Incident CHD event (before age 75)	261 (7%)	324 (7%)	172 (4%)	757 (6%)	173 (18%)	414 (17%)	587 (17%)

Categorical variables are shown as counts and percentages, continuous variables (age, follow-up time, cholesterol, and blood pressure) as means and standard deviations. Sample sizes are for participants with GWAS data after quality control and all other exclusions. Lipid lowering treatments were not assessed in FHS due to an insufficient number of exams with this information.

The outcome of interest in FINRISK was primary incident CHD event, defined as myocardial infarction (MI), a coronary revascularization procedure, or death from CHD, before age 75 years (see Supplementary Data online, *Supplementary Methods*). Individuals with prevalent cardiovascular disease (CVD) at baseline were excluded from the analysis. We censored events for individuals with an attained age of >75 years, as not all FINRISK cohorts had sufficient numbers of CHD events beyond that age. In FHS, we used the FHS definition of CHD, which included recognized/unrecognized MI or death from CHD as well as angina pectoris or coronary insufficiency (see Supplementary Data online, *Supplementary Methods*). FHS individuals with prevalent CHD or <30 years of age at baseline were excluded, and for consistency with the FINRISK analysis, a censoring age of 75 years was also applied to the FHS analyses.

Secondary external validation of the GRS was also performed in the ARGOS study, a Dutch case/control dataset where all individuals had familial hypercholesterolemia (248 young cases with early CHD, 216 elderly controls without CHD), imputed to 1000 Genomes reference panel (74 135 SNPs of the 79 128 CARDIoGRAMplusC4D SNPs were available; see Supplementary Data online, *Supplementary Methods*).

### Statistical analysis

GRSs were generated via thinning the CARDIoGRAMplusC4D SNPs by linkage disequilibrium (LD) thresholds and evaluated using logistic regression and area under receiver-operating characteristic curve (AUC) for each threshold (see Supplementary Data online, *Figure S1*). To avoid overfitting we only used weights (log odds) from the CARDIoGRAMplusC4D stage-2 meta-analysis, which were not based on the WTCCC-CAD or MIGen studies (see Supplementary Data online, *Supplementary Methods*). We combined the estimates for WTCCC and MIGen-Harps using fixed-effects inverse-variance weighted meta-analysis.

Subsequent performance of the GRS was evaluated in external, independent validation data. For analysis of FINRISK, we used Cox proportional hazard models to evaluate the association of the GRS with time to incident CHD events, stratifying by sex and adjusting for geographic location and cohort, using age as the time scale. Secondary analyses adjusted for one of the clinical risk scores (FRS or ACC/AHA13), or individual baseline variables and known risk factors (cohort, geographical location, prevalent type-2 diabetes, log total cholesterol, log HDL, log systolic BP, smoking status, lipid treatment, and family history). Family history in FINRISK was self-reported and was defined as having a 1st-degree relative who had experienced MI before age 60. For FHS, we evaluated the association of the GRS with incident CHD using Cox proportional hazard models, stratifying by sex and adjusting for cohort (Original or Offspring), using age as the time scale. Family history was not available for both FHS cohorts and thus not considered in FHS analyses. Survival analyses allowing for competing risks were performed using the Aalen-Johansen estimator of survival and cause-specific Cox models (see Supplementary Data online, *Supplementary Methods***).** Model discrimination of incident CHD event was evaluated in three groups of individuals: (i) all individuals (*n* = 12 676 in FINRISK, *n* = 3406 in FHS), (ii) individuals aged <60 years at baseline (*n* = 10 606 in FINRISK, *n* = 3218 in FHS), and (iii) individuals aged ≥60 years at baseline (*n* = 2070 in FINRISK, *n* = 188 in FHS).

Discrimination of incident CHD events within 10 years was assessed using Harrell’s C-index, and the difference in C-index between two models was assessed using the correlated jackknife test. Competing risk analyses were performed using the Aalen-Johansen empirical estimator of cumulative incidence and cause-specific Cox proportional hazard models. Risk reclassification was evaluated using continuous Net Reclassification Improvement (NRI), categorical NRI, and Integrated Discrimination Improvement. Meta-analysis of the discrimination statistics was performed using fixed-effect inverse-variance weighting. Additional details on the statistical methods are provided in the see Supplementary Data online, *Supplementary Methods*.

## Results

To construct an optimized GRS using the WTCCC and MIGen-Harps datasets, we first generated a series of GRSs, starting with the 79 128 CARDIoGRAMplusC4D SNPs then progressively lowering the *r*^2^ threshold for LD to reduce the redundancy of predictive information and corresponding number of SNPs in the score (Methods and *Figure 1*). An *r^2^* threshold of 0.7 provided optimal discrimination of CHD cases and controls (WTCCC and MIGen-Harps meta-analysis odds ratio(OR) = 1.70 per S.D. of GRS, 95% confidence interval (CI 1.61–1.80; meta-analysis AUC = 0.64, 95% CI 0.63–0.66), corresponding to 49 310 SNPs in WTCCC (see Supplementary Data online, *Figure S1*). Of these 49 310 SNPs, 85.9% (42 364 SNPs) and 95% (46 773 SNPs) were available in the FINRISK and FHS, respectively.

The 49K GRS showed similar odds ratios for incident CHD as a binary outcome in FINRISK (OR = 1.74, 95% CI 1.61–1.89, per S.D.), WTCCC (OR = 1.74, 95% CI 1.63–1.86, per S.D.), and MIGen-Harps (OR = 1.57, 95% CI 1.37–1.81, per S.D.) (*Table 2*). However in the FHS, the association was weaker, OR = 1.30 (95% CI 1.19–1.43, per S.D.) (*Table 2*). Density plots of the GRS in FINRISK and FHS for those with and without CHD <75 years are shown in see Supplementary Data online, *Figure S2*.

**Table 2:** Association of the 49K GRS with incident CHD (binary outcome in logistic regression) in the five studies, per standard deviation of the GRS

Dataset	# Incident CHD/Non-CHD	Odds Ratio (95% CI)
WTCCC-CAD1	1926/2938	1.74 (1.63–1.86)
MIGen-Harps	488/531	1.57 (1.37–1.81)
ARGOS FH	248/216	1.49 (1.21–1.84)
FINRISK	757/11919	1.74 (1.61–1.89)
FHS	587/2819	1.28 (1.17–1.41)

WTCCC-CAD1: adjusted for sex and 5 PCs of the genotypes; MIGen-Harps: adjusted for sex and 5 PCs; ARGOS: adjusted for sex and 5 PCs; FINRISK: adjusted for sex, cohort, east/west, and 5 PCs; FHS: adjusted for sex, cohort, and 5 PCs.

Using survival analyses of time to incident CHD, within FINRISK the GRS had stronger association with CHD (HR = 1.74, 95% CI 1.61–1.86, per S.D.) than the 28 SNP score studied by Tikkanen *et al.*^11^ (HR = 1.21, 95% CI 1.13–1.30, per S.D.), the 27 SNP score used by Mega *et al.*^29^ (HR = 1.21, 95% CI 1.12–1.30 per S.D.), or the 153 SNPs found at FDR <0.05 by the CARDIoGRAMplusC4D consortium^8^ (HR = 1.25, 95% CI 1.16–1.39 per S.D.) (see Supplementary Data online, *Supplementary Results*). In FHS, the GRS showed weaker but statistically significant association with CHD (HR = 1.28 per S.D. of the GRS, 95% CI 1.18–1.38). The fixed-effect meta-analysis estimate for the GRS combining FINRISK and FHS was HR = 1.66 (95% CI 1.55–1.78), however, heterogeneity was high (*I*^2 ^=^ ^89.2%, Cochran’s *Q P *=* *0.0023). The top vs. bottom quintiles of the GRS showed significantly different incident CHD risk overall (FINRISK HR = 4.51, 95% CI 3.47–5.85; FHS HR = 1.84 95% CI 1.43–2.37). For both FINRISK and FHS, the GRS showed improved prediction for incident CHD over the other risk scores composed of smaller numbers of SNPs (see Supplementary Data online, *Supplementary Results and Table S3*).

In both FINRISK and FHS, the hazard ratios for GRS were not substantially attenuated by adjusting for FRS or ACC/AHA13 clinical risk scores, lipid treatment at baseline, other established risk factors (including family history in FINRISK), or 5 principal components of the genotypes (see Supplementary Data online, *Figures S3 and S4*). The correlation between GRS and either FRS or ACC/AHA13 scores was close to zero with almost none of the variation in GRS explained by either clinical risk score (in both FINRISK and FHS, *r*^2 ^<^ ^0.004 between GRS and either FRS and ACC/AHA13; see Supplementary Data online, *Figure S5*). To further test that the CHD risk conferred by the GRS was largely independent of the effects of cholesterol, we further validated the GRS in the ARGOS familial hypercholesterolemia study, with comparable results to those obtained in WTCCC/MIGen (OR = 1.49, 95% CI 1.21–1.84 per S.D. of the GRS, adjusted for sex and five principal components) (see Supplementary Data online, *Supplementary Methods*).

**Figure 2:**
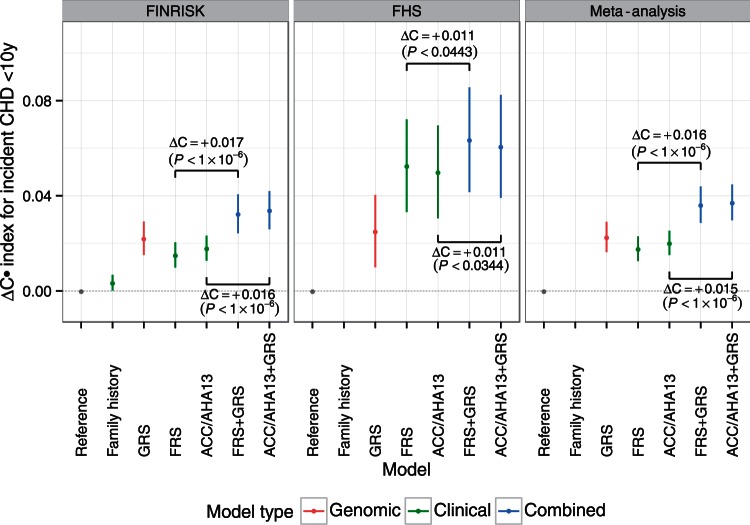
Difference in C-index (95% CI) for time to incident CHD event within 10 years, relative to the reference model in the FINRISK and FHS cohorts. Reference models used age as the time scale, stratified by sex (FINRISK: adjusted for cohort and geographic location; FHS: adjusted for cohort). Family history was not available for all of the FHS cohorts and thus not considered here. *P*-values are from the correlated jackknife test.

To assess the predictive power of the GRS, we compared its performance in discrimination of time to CHD event (C-index) with that of family history and the FRS and ACC/AHA13 clinical risk scores. We also assessed the incremental value of the GRS on top of the clinical risk scores. In both FINRISK and FHS, addition of GRS to either FRS or ACC/AHA13 scores provided statistically significant improvements in C-index, in FINRISK: +1.7% (*P *<* *10 ^−^ ^6^) and +1.6% (*P *<* *10 ^−^ ^6^) for FRS and ACC/AHA13, respectively; in FHS: +1.1% (*P *<* *0.0443) and +1.1% (*P *<* *0.0344) for FRS and ACC/AHA13, respectively (*Figure 2*). Overall, fixed-effects meta-analysis of the two studies showed that GRS improved the C-index by +1.6% (95% CI 0.01–0.02, *P *<* *10 ^−^ ^6^; heterogeneity: *I*^2 ^=^ ^2.2%, *Q *=* *1.02, *P *=* *0.312) for FRS and GRS combined (FRS + GRS) over FRS alone and, similarly, +1.5% (95% CI 0.009–0.02, *P *<* *10 ^−^ ^6^; heterogeneity: *I*^2 ^=^ ^0%, *Q *=* *0.78, *P *=* *0.378) for ACC/AHA13 + GRS over ACC/AHA13 alone (*Figure 2*). Larger increases in C-index were observed among older individuals, with the C-index of FRS + GRS compared with FRS alone increasing by 5.1% in individuals aged ≥60 years at baseline, while individuals aged <60 years at baseline showed C-index gains of 1.4% (see Supplementary Data online, *Figure S6*). Within FINRISK, the GRS had higher C-index than family history (+1.9%, *P *<* *1.3 × 10 ^−^ ^6^).

We assessed if the GRS improved the individual 10 years risk reclassification when added to clinical risk scores. Analyses within FINRISK and FHS are given in *Table 3* for FRS and in *Table 4* for ACC/AHA13. Overall, meta-analysis of the two datasets showed that the categorical Net Reclassification Improvement was 0.1 for both FRS + GRS and ACC/AHA13 + GRS, respectively (*P *<* *0.0001; see Supplementary material online, *Figure S7*). Meta-analysis of continuous NRI was 0.344 (*P *<* *0.001) and 0.334 (*P *<* *0.001) for the FRS + GRS and ACC/AHA13 + GRS, respectively **(**see Supplementary Data online, *Figure S8*). Meta-analysis of IDI scores showed gains of 0.01 (*P *<* *0.001) and 0.009 (*P *<* *0.001) for FRS + GRS and ACC/AHA13 + GRS, respectively, however IDI scores showed high heterogeneity across FINRISK and FHS (*I*^2 ^>^ ^97%, Cochran’s *Q P *<* *0.0001, see Supplementary Data online, *Figure S9*).

**Table 3:** Reclassification of incident CHD event risk within 10 years for combined FRS + GRS compared with FRS only, in the FINRISK and FHS cohorts

		FINRISK						FHS					
		FRS+GRS						FRS+GRS					
		0–7.5%	7.5–10%	10–20%	20–100%	Total	Reclass %	0–7.5%	7.5–10%	10–20%	20–100%	Total	Reclass %
All individuals													
FRS	0–7.5%	9566	218	138	6	9928	3.6	2482	88	4	0	2574	3.6
	7.5–10%	368	190	223	21	802	76.3	122	165	83	1	371	55.5
	10–20%	299	290	767	298	1,654	53.6	11	74	339	19	443	23.5
	20–100%	1	14	114	156	285	15.7	0	0	5	13	18	27.8
	Total	10,234	712	1,242	481	12669	15.7	2,615	327	431	33	3,406	11.9

Incident CHD present													

FRS	0–7.5%	110	21	19	2	152	27.6	67	6	0	0	73	8.2
	7.5–10%	22	12	28	4	66	81.8	5	11	6	0	22	50.0
	10–20%	22	24	108	78	232	53.4	2	5	43	4	54	20.4
	20–100%	0	2	17	48	67	28.4	0	0	0	1	1	0
	Total	154	59	172	132	517	46.2	74	22	49	5	150	18.7

Incident CHD absent													

FRS	0–7.5%	9456	197	119	4	9776	3.3	2415	82	4	0	2501	3.4
	7.5–10%	346	178	195	17	736	75.8	117	154	77	1	349	55.9
	10–20%	277	266	659	220	1422	53.7	9	69	296	15	389	23.9
	20–100%	1	12	97	108	218	50.5	0	0	5	12	17	29.4
	Total	10 080	653	1070	349	12 152	14.4	2541	305	382	28	3256	11.6

All individuals													
		FINRISK						FHS				
		FRS+GRS						FRS+GRS
NRI (categorical) [95% CI]		Total: 0.146 [0.088–0.20]; *P* < 1 × 10^−6^ NRI for events: 0.126 [0.068–0.183]; *P* = 1.9 × 10^−5^ NRI for non-events: 0.020 [0.014–0.027]; *P* < 1 × 10^−6^						Total: 0.033 [−0.037–0.103]; *P* = 0.35 NRI for events: 0.27 [−0.042–0.096]; *P* = 0.449 NRI for non-events: 0.006 [−0.005–0.018]; *P* = 0.281				
NRI (continuous) [95% CI]		Total: 0.371 [0.285–0.457]; *P* < 1 × 10^−6^ NRI for events: 0.195 [0.111–0.280]; *P* < 6 × 10^−6^ NRI for non-events: 0.175 [0.159–0.192]; *P* < 1 × 10^−6^						Total: 0.249 [0.087–0.411]; *P* < 0.0026 NRI for events: 0.147 [−0.012–0.305]; *P* = 0.069 NRI for non-events: 0.102 [0.069–0.136]; *P* < 1 × 10^−6^				
IDI (continuous) [95% CI]		0.028 [0.026–0.034]; *P* < 1 × 10^−6^						0.005 [0.002–0.008]; *P* < 0.00098				

In FINRISK, 7 individuals of the 12 676 were excluded in this analysis due to missing clinical measurements.

**Table 4:** Reclassification of incident CHD event risk within 10 years for combined ACC/AHA13 + GRS compared with ACC/AHA13 only, in the FINRISK and FHS cohorts

		FINRISK						FHS					
		ACC/AHA13+GRS						ACC/AHA13+GRS					
		0–7.5%	7.5–10%	10–20%	20–100%	Total	Reclass %	0–7.5%	7.5–10%	10–20%	20–100%	Total	Reclass %
All individuals													
ACC/AHA13	0–7.5%	9,588	211	144	7	9,950	3.6	2,513	78	7	0	2,598	3.3
	7.5–10%	381	176	199	14	770	77.1	112	159	66	1	338	53.0
	10–20%	279	275	755	271	1,580	52.2	7	67	308	32	414	25.6
	20–100%	2	10	127	230	369	37.7	0	0	16	40	56	28.6
	Total	10,250	672	1,225	522	12,699	15.2	2,632	304	397	73	3,406	11.3

Incident CHD present													
ACC/AHA13	0–7.5%	118	16	17	1	152	22.4	67	8	0	75	75	10.7
	7.5–10%	20	14	29	6	69	79.7	6	6	11	23	23	73.9
	10–20%	15	29	104	60	208	50.0	1	6	34	46	46	26.1
	20–100%	0	0	15	73	88	17.0	0	0	2	6	6	33.3
	Total	153	59	165	140	517	40.2	74	20	47	150	150	26.0

Incident CHD absent													
ACC/AHA13	0–7.5%	9,470	195	127	6	9,798	3.3	2,446	70	7	0	2,523	3.1
	7.5–10%	361	162	170	8	701	76.9	106	153	55	1	315	51.4
	10–20%	264	246	651	211	1,372	62.6	6	61	274	27	368	25.5
	20–100%	2	10	112	157	281	44.1	0	0	14	36	50	28.0
	Total	10,097	613	1,060	382	12,152	14.1	2,558	284	350	64	3,256	10.7

All individuals													
	FINRISK						FHS					
	ACC/AHA13+GRS						ACC/AHA13+GRS					
NRI (categorical) [95% CI]	Total: 0.120 [0.065–0.174]; *P* = 1.7 × 10^−5^ NRI for events: 0.097 [0.043–0.151]; *P* = 4.52 × 10^−4^ NRI for non-events: 0.023 [0.016–0.030]; *P* < 1 × 10^−6^						Total: 0.068 [−0.014–0.150]; *P* = 0.1 NRI for events: 0.060 [−0.021–0.141]; *P* = 0.147 NRI for non-events: 0.008 [−0.003–0.020]; *P* = 0.147					
NRI (continuous) [95% CI]	Total: 0.356 [0.270–0.442]; *P* < 1 × 10^−6^ NRI for events: 0.176 [0.091–0.261]; *P* = 4.79 × 10^−5^ NRI for non-events: 0.180 [0.164–0.196]; *P* < 1 × 10^−6^						Total: 0.255 [0.093–0.416]; *P* = 0.00197 NRI for events: 0.160 [0.002–0.318]; *P* = 0.047 NRI for non-events: 0.095 [0.061–0.128]; *P* < 1 × 10^−6^					
IDI (continuous) [95% CI]	0.028 [0.021–0.034]; *P* < 1 × 10^−6^						0.005 [0.002–0.008]; *P* = 0.00184					

In FINRISK, 7 individuals of the 12,676 were excluded in this analysis due to missing clinical measurements.

**Figure 3:**
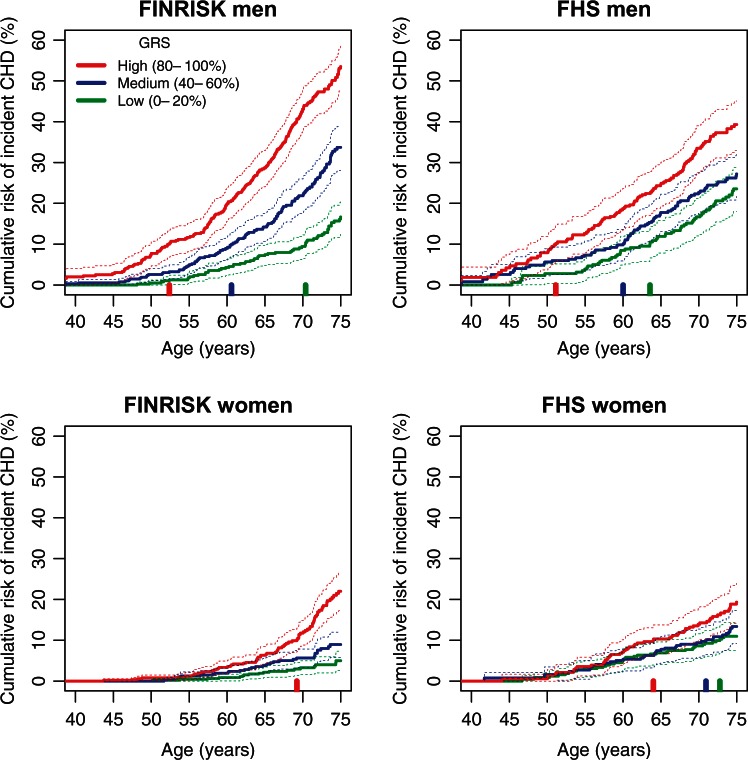
Kaplan-Meier cumulative risk of incident CHD event by genomic risk group for men and women in the FINRISK and FHS cohorts. Showing the cumulative risk in quintiles 0–20%, 40–60%, 80–100%. The vertical bars along the x-axis indicate the age at which each risk group attains a cumulative CHD risk of 10%. Dashed lines indicate 95% CI.

We next examined how variation in genomic risk translated into differences in cumulative lifetime risk of CHD, using Kaplan-Meier estimates stratified by GRS quintiles for men and women separately (*Figure 3*). As expected, cumulative risk increased with age for both sexes, with men displaying higher absolute risk than women. In both sexes there were substantial differences in cumulative risk between GRS groups with 1.7-fold (in FHS) to 3.2-fold (in FINRISK) higher cumulative risk by age 75 in those in the top quintile of GRS vs. bottom quintile. When considering clinically relevant levels of risk, FINRISK men in the top quintile of genomic risk achieved 10% cumulative risk 18 years earlier than those in the bottom quintile (ages 52 and 70, respectively), with a comparable difference of 12 years in FHS (ages 51 and 64). Women in the top quintile of genomic risk achieved 10% cumulative risk by age 69 (FINRISK) and 64 (FHS), whereas women in the bottom quintile did not achieve 10% risk by age 75 in FINRISK, or by age 73 in FHS. Estimated lifetime CHD risk in FINRISK showed no evidence of being affected by competing risks (incident CHD vs. non-CHD death) (see Supplementary Data online, *Supplementary Methods* and Supplementary Figure S10). Similarly, a cause-specific competing-risk Cox analysis of the GRS in FINRISK, adjusting for geographical location and cohort, resulted in a similar hazard ratio as standard Cox analysis (HR = 1.70, 95% CI 1.61–1.86).

**Figure 4:**
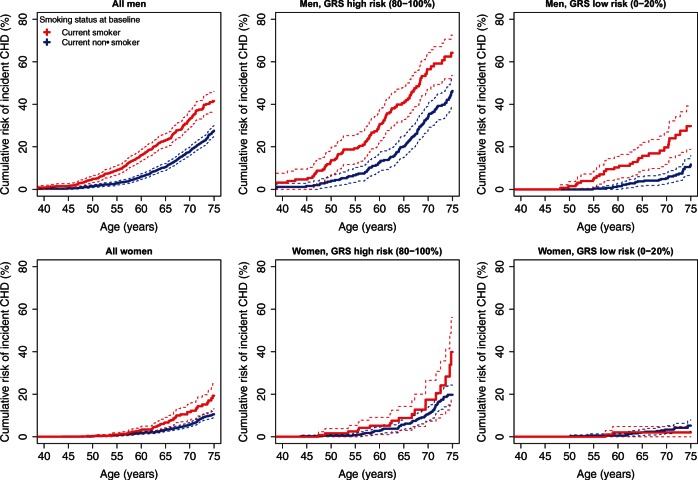
Kaplan-Meier curves for incident CHD event risk stratified by GRS quintiles and smoking status at baseline, for men and women in the FINRISK cohorts.

We next sought to investigate to what degree high genomic risk for CHD could be compensated for by low levels of clinical risk factors at baseline, and vice-versa. When considering baseline smoking status in both FINRISK and FHS, Kaplan-Meier analysis showed a substantial increase in cumulative risk of CHD in men who smoked and were also in the top quintile of genomic risk, relative to either non-smokers or smokers at low genomic risk (*Figure 4* for FINRISK and see Supplementary Data online, *Figure S11* for FHS). Similar but weaker trends were observed for women in the top vs. bottom quintiles of genomic risk. To test whether there was evidence for smoking affecting CHD hazard differently based on an individual’s genomic background, we used a Cox model allowing for an interaction term between the GRS and smoking; the interaction was not statistically significant in FINRISK (*P *=* *0.91) and FHS (*P *=* *0.49).

We also examined the potential compensatory effects of baseline systolic blood pressure and total cholesterol, divided as tertiles of high, medium, and low levels (see Supplementary Data online, *Figures S12 and S13*). For both systolic blood pressure and total cholesterol, we observed the expected trends in CHD risk for high, medium and low levels. However, males with high vs. low levels of systolic blood pressure or total cholesterol showed greater absolute CHD risk if they were in the top vs. bottom quintiles of genomic risk.

Notably, in both FINRISK and FHS, women in the bottom quintile of genomic risk showed smaller differences in cumulative CHD risk when stratified by smoking. For tertiles of systolic blood pressure or total cholesterol, low genomic risk women in FINRISK showed similarly small differences in risk, but the effects in FHS for this subgroup were not consistent. Cox models allowing for interactions between the GRS and systolic blood pressure or total cholesterol did not show statistically significant interactions in either FINRISK or FHS (*P *>* *0.2 for all).

## Discussion

We have generated a GRS for CHD based on 49 310 SNPs and, using three prospective FINRISK and two FHS prospective cohorts, demonstrated that the GRS is associated with incident CHD events independently of established and widely-used clinical risk scores or individual CHD risk factors, including family history. Secondary validation in a familial hypercholesterolemia study (ARGOS) showed that GRS was also associated with CHD in this group of high-risk individuals. Subsequently, combining the GRS with established risk scores improved 10-year CHD risk prediction in FINRISK and FHS. We have also shown that the GRS can be leveraged to achieve meaningful lifetime CHD risk stratification, and that the impact of traditional CHD risk factors such as smoking, blood pressure, and cholesterol, vary substantially depending on the underlying genetic risk, thus offering the potential for both earlier and more targeted preventative efforts.

A distinctive feature of our analysis compared with several previous prospective studies^11^^,^^29^^,^^30^ examining the predictive utility of GRS for incident CHD is that the best predictive model was achieved here with SNPs that did not necessarily reach genome-wide or even statistical significance in previous GWA studies. The GRS outperformed other smaller SNP models, and shows greater promise in CHD prediction between top and bottom GRS quintiles than a recently published study testing a genetic risk score of 50 SNPs in Scandinavians^30^ (GRS50 HR = 1.92 vs. GRS49K HR = 4.51). Genome-wide SNP models have been applied successfully to other heritable human traits which seem to follow an “infinitesimal” genetic architecture, such as height.^18^ These results highlight the differing goals of GWAS and of genomic prediction: the stringent detection of causal genetic variants involved in the disease process vs. the construction of a model that robustly and maximally predicts future disease. While stringent procedures for minimizing the false positive rate of associated loci in GWAS are appropriate, these concerns are less relevant in construction of GRSs, especially when there are a large number of weakly correlated SNPs^20^ and when rigorous internal and external validation is performed.

While population stratification is a potential confounder of genomic prediction studies, our use of a large worldwide multi-ethnic meta-analysis to develop the GRS together with two fully independent prospective validation datasets and three independent case/control datasets minimizes this potential. Our GRS was constructed from the CARDIoGRAMplusC4D stage-2 meta-analysis and the FINRISK and FHS individuals are both independent of that study and of broadly European ancestry; thus it is unlikely that the GRS is substantially confounded by fine-scale population structure within these cohorts. Further, the LD-thinning threshold to maximize prediction was determined in the WTCCC and MIGen datasets prior to applying the GRS to ARGOS, FINRISK, or FHS. Nevertheless, for some measures, GRS gains were less pronounced in FHS than in FINRISK. This may partly be due to the different definitions of CHD in these studies, to differences in environmental exposures, or to differences in genetic effects.^31^ In addition, the FRS was developed in the FHS, leading to potential over-estimation of its association with CHD in the current analysis. Hence, there may be benefit from future development of population-specific GRSs, which may yield greater predictive power within each population.

The association of the GRS with incident CHD was not substantially attenuated by traditional risk factors or clinical risk scores derived from these risk factors. Furthermore, the GRS was strongly associated with CHD in a study consisting purely of individuals with familial hypercholesterolemia. These results suggest that genomic risk exerts its effect on CHD risk through molecular pathways that are largely independent of the effects of cholesterol, systolic blood pressure, and smoking. A hitherto unresolved question has been the extent to which a family history would capture any information that may be provided through genetic analysis. Here, we clearly demonstrate the superior performance of direct genetic information over self-reported family history of CHD, which is often incomplete and imprecise in practice and is influenced by family size and competing causes of death.

While we observed improvements in discrimination (C-index) resulting from adding the GRS to the clinical risk scores when considering adults of all ages, the improvements were substantially higher in older individuals (>60 years old). Rather than being driven by age-related differences in the effect of the GRS, these results are likely driven by differences in the clinical risk scores between the younger and older adults. Unlike the GRSs, the clinical risk scores showed substantial differences across ages, driven by temporal changes in the underlying risk factors as well as age itself. Beyond the aims of identifying older adults with high CHD risk, the invariance of genomic risk makes it particularly useful for CHD risk prediction earlier in life, in young adulthood or before, when traditional risk factors are typically not measured and less likely to be informative of risk later in life.

Our analyses focused on two clinical scores, the FRS and ACC/AHA13. While other scores exist, for example the SCORE system,^32^ we elected to use the FRS and ACC/AHA13 due to their widespread use and the fact that the FINRISK cohorts were a major contributor to the SCORE analysis, potentially biasing the analysis in FINRISK, in the same way that FRS seems to be biased towards the FHS, inflating its predictive power of the clinical risk scores there relative to the reference model.

Stratifying individual baseline smoking, systolic blood pressure, and total cholesterol levels measures into genomic risk groups revealed substantial differences in cumulative risk patterns. Importantly, this demonstrates that improved lifestyle may compensate for the innate increased CHD risk captured by the GRS. For men with high genomic risk, modifiable risk factors showed large effects on cumulative CHD risk. For women, the observed impacts of smoking, systolic blood pressure, and total cholesterol were low or not detectable in the low genomic risk group, particularly in FINRISK, however, we could not determine whether this was due to inadequate statistical power or other biological effects and further studies in larger cohorts of women are necessary to determine any clinical implications.

Our results, if validated in further studies and across different populations, suggest a potential paradigmatic shift in the current CHD screening strategy which has existed for over 40 years—namely determination of genomic risk at an early stage with screening later in life through traditional clinical risk scores to complement background genomic risk. Based on early genomic risk stratification, individuals at higher risk may benefit from earlier engagement with nutritionists, exercise regimes, smoking cessation programs or be initiated early on medical interventions such as statin therapy or blood-pressure lowering medications to minimize future CHD risk. In this context it is notable that Mega *et al.*^29^ recently demonstrated that the GRS of 27 CHD-associated SNPs better predicted which individuals would benefit most, both in relative and absolute terms, from statin treatment. In a study of type 2 diabetes, Florez *et al.*^32^ has shown that the effects of increased genetic susceptibility to disease can be ameliorated by lifestyle (diet and exercise) and therapeutic (metformin) interventions. Similar possibilities exist for CHD, whereby early targeted prevention strategies based on genomic CHD risk may be implemented well in advance of clinical risk scores attaining predictive capacity at later ages.^33^ Such early risk stratification will offer increased efficiency in allocating both therapeutic resources and lifestyle modifications with the potential for subsequent delay of onset of traditional risk factors and incident CHD risk.

While our study demonstrates both the independent and incremental predictive power provided by our GRS, it is important to note that even when combined with such scores, the overall positive predictive value still remains modest for an acceptable negative predictive value (see Supplementary Data online, *Figure S14a*). Furthermore, despite overall improved reclassification of 10 years risk, some individuals who went on to develop an incident event were reclassified at a lower risk by the addition of the GRS compared with their initial classification using a clinical score (*Tables 3 and 4*), emphasizing the limitations of the current GRS. The magnitude of the GRS effect was weaker in FHS than in the other datasets examined (FINRISK, WTCCC-CAD, MIGen-Harps, and ARGOS; *Table 2*). In addition to potential technical and clinical FHS differences discussed above, these results suggest that the benefit and clinical utility of the GRS may vary between populations; further evaluation in large prospective studies of varying ancestry will be required in order to assess these differences and how best to account for them in risk prediction. In this context, it should be noted that our GRS based on a starting list of 79 128 common SNPs tested by the CARDIOGRAMplusC4D consortium could be further improved. Future studies that construct GRSs using increased sample sizes and capturing the full spectrum of common and rare variants^9^^,^^34^ will likely provide additional gains in prediction and risk stratification.

In summary, this study has demonstrated the potential clinical utility of genome-scale GRS for CHD, both for early identification of individuals at increased CHD risk and for complementing existing clinical risk scores. Given recent advances and reduced cost of genotyping microarrays and sequencing-based technologies and their cost efficiency, determination of genome-wide SNP variants (including the 49 310 SNPs used here) is no longer beyond the realm of clinical application. In terms of technical feasibility, genome-wide genotyping of hundreds of thousands of SNPs is now both reliable and cost effective (<US$70 in bulk), and clinically certified genotyping services are now becoming available. Statistical SNP imputation will further expand the number of SNPs to an order of several million. Additionally, germline genotyping is a one-time cost for each individual. Further validation and cost-benefit analyses will be required in order to establish how this technology is deployed in clinical settings.

## Supplementary Material

Supplementary Data

Supplementary Data
