# Supplementary material for: Genomic prediction of coronary heart disease
Source: Eur Heart J. 2016 Sep 21;37(43):3267–78. doi: 10.1093/eurheartj/ehw450 (PMC5146693; doi:10.1093/eurheartj/ehw450)
Supplement: Supplementary Data [file ehw450_supplementary_data.zip › ehw450_Supp.docx]

**Supplementary Appendix**

**Genomic prediction of coronary heart disease**

Gad Abraham PhD^1,2^, Aki S. Havulinna DSc^3^, Oneil G. Bhalala PhD^1,2^, Sean G. Byars PhD^1,2^_,_ Alysha M. De Livera PhD^1,2,4^, , Laxman Yetukuri PhD^5^, Emmi Tikkanen PhD^5^, Markus Perola MD PhD^3,5^, Heribert Schunkert MD^6^, Eric J. Sijbrands MD PhD^7^, Aarno Palotie MD PhD^5,8,9,10^, Nilesh J. Samani MD FRCP^11,12,#^, Veikko Salomaa MD PhD^3,#^, Samuli Ripatti PhD^5,13,14,*,#^, Michael Inouye PhD^1,2,5,*,#^

^1^ Centre for Systems Genomics, School of BioSciences, The University of Melbourne, Parkville 3010, Victoria, Australia

^2^ Department of Pathology, The University of Melbourne, Parkville, Victoria 3010, Australia

^3^ National Institute for Health and Welfare, Helsinki, Finland

^4^ Centre for Epidemiology and Biostatistics, Melbourne School of Population and Global Health, The University of Melbourne, Parkville, Victoria 3010, Australia

^5^ Institute for Molecular Medicine Finland (FIMM), University of Helsinki, Helsinki, Finland

^6^ Deutsches Herzzentrum München, Klinik für Herz- und Kreislauferkrankungen, Munich, Germany

^7^ Department of Internal Medicine, Erasmus Medical Center, Rotterdam 3000 CA, The Netherlands

^8^Analytic and Translational Genetics Unit, Department of Medicine, Massachusetts General Hospital, Boston, Massachusetts, USA

^9^ Program in Medical and Population Genetics, Broad Institute of Harvard and MIT, Cambridge, Massachusetts, USA

^10^ Psychiatric & Neurodevelopmental Genetics Unit, Department of Psychiatry, Massachusetts General Hospital, Boston, Massachusetts, USA

^11^ Department of Cardiovascular Sciences, University of Leicester, BHF Cardiovascular Research Centre, Glenfield Hospital, Groby Rd., Leicester, LE3 9QP, United Kingdom

^12^ National Institute for Health Research Leicester Cardiovascular Biomedical Research Unit, Glenfield Hospital, Groby Road, Leicester, LE3 9QP, United Kingdom

^13^ Wellcome Trust Sanger Institute, Wellcome Trust Genome Campus, Hinxton, Cambridge, United Kingdom

^14^ Department of Public Health, University of Helsinki, Helsinki, Finland

* These authors contributed equally

^#^ Corresponding authors: Michael Inouye (minouye@unimelb.edu.au), Samuli Ripatti (samuli.ripatti@helsinki.fi), Veikko Salomaa (veikko.salomaa@thl.fi), and Nilesh J. Samani (njs@leicester.ac.uk)

**Study Design**

This study consisted of two main stages (**Figure 1** in the main text). In stage 1, we utilized the large-scale CHD genetic association dataset assembled by the CARDIoGRAMplusC4D consortium^1^ (downloaded from http://www.cardiogramplusc4d.org) to construct a GRS. Briefly, CARDIoGRAMplusC4D tested associations of 79,128 SNPs in 63,746 CHD cases and 130,681 controls, including 6,222 SNPs that had shown nominal association with CHD in a prior GWAS meta-analysis^2^. We extracted information on all these SNPs together with their associated CARDIoGRAMplusC4D weights (log odds) and used two case/control datasets, the Wellcome Trust Case/Control Consortium Coronary Artery Disease dataset (WTCCC-CAD)^3^ (1,926 cases and 2,938 controls) and the MIGen case/control dataset^4^ (Harps subset) (531 cases and 488 controls), to optimize the predictive value of this set of SNPs using linkage-disequilibrium (LD) thinning to derive a GRS. In stage 2, we assessed the predictive accuracy of this GRS with incident CHD events in the FINRISK and the Framingham Heart Study cohorts, comparing it to traditional risk factors and clinical risk scores. Secondary validation was also performed in the ‘Association of CHD Risk in a Genome-wide Old-versus-young Setting’ (ARGOS) study^5^ familial hypercholesterolemia study. Details of the final GRS used are available at <http://www.inouyelab.org>.

**Supplementary Results**

**Association of GRS with CHD in ARGOS**

We performed logistic regression of case/control CHD status on the GRS in 1000 Genomes imputed ARGOS data (n=464 individuals), adjusting for sex and 5 principal components (PCs, **Supplementary Methods**). The GRS had an association of OR=1.49 (95% CI 1.21–1.84, per S.D. of the GRS), and AUC =0.61 (95% CI 0.56–0.66). The reference model (sex and 5 PCs) had an AUC = 0.59 (95% CI 0.54–0.64).

**Association results for other genomic risk scores**

For the Tikkanen 28-SNP score ^6^, 28 SNPs were available for FINRISK batch I (n=7,050 individuals) and 9 for FINRISK batch II (n=5626 individuals) (see **Supplementary Methods** for information about the genotyping batches). The Tikkanen score had an association of HR=1.24 (95% CI 1.15–1.34, per S.D. of the score) in batch I, and HR=1.22 (95% CI 1.13–1.30, per S.D. of the score) in the combined data. In FHS, all 28 SNPs were available, and the association was HR=1.15 (95% CI 1.06–1.25, per S.D. of the score).

For the Mega score ^7^, 26 of the 27 SNPs were available for FINRISK batch I and 12 SNPs for batch II. In analysis of batch I only (n=7,046 individuals with non-missing clinical variables), the score had an association of HR=1.21 (95% CI 1.12–1.30, per S.D. of the score), and HR=1.22 (95% CI 1.13–1.30, per S.D. of the score) in the combined data. In FHS, 26 of the SNPs were available, and the association was HR=1.20 (95% CI 1.07–1.26, per S.D. of the score).

For the 153 SNP identified by CARDIoGRAMplusC4D at FDR <0.05, 151 of 153 SNPs mapped to FINRISK batch I and 101 to batch II. In batch I, the association was HR = 1.25 (1.16–1.39) per S.D. of the score. In the combined FINRISK dataset, the association was HR = 1.26 (1.18–1.36) per S.D. of the score. In FHS, 153 SNPs mapped, and the association was HR = 1.21 (1.16–1.32) per S.D. of the score.

Comparisons of the improvement in 10y C-index for the GRS over the other three genomic scores are given in **Table S3**. We performed the comparison only in batch I to avoid confounding by the differential missingness of the three scores, which was substantial as a proportion of the number of SNPs in each score.

**C-index improvements within age groups**

Using the same Cox models developed on all individuals (n=12,676 in FINRISK and 3,406 in FHS), we predicted the absolute risk of CHD <10y from baseline in all individuals, and subsequently examined the concordance (C-index) within all individuals and two subgroups: individuals <60yo at baseline, and individuals ≥60yo at baseline. In meta-analysis, the improvements in C-index for the younger individuals were lower than those for the older individuals (**Figure S6**). Adding the GRS to the clinical risk scores improved discrimination by +1.4% (*P*=2.2×10^-5^) for FRS (**Figure S6a**) and +1.3% for ACC/AHA13 (*P*=2×10^-5^) (**Figure S6c**). For older individuals (≥60 years old at baseline), adding the GRS to the clinical risk scores increased discrimination over the FRS (+5.1%, *P*=2.6×10^-6^) (**Figure S6b**) and the ACC/AHA13 score (+4.6%, *P*=1.5×10^-5^) (**Figure S6d**). Further investigation of differences in performance between younger and older age groups in FINRISK revealed no differences in FRS, ACC/AHA13, or GRS effects over time (*P*=0.3, *P*=0.1, and *P*=0.1; **Supplementary Methods**), however as expected there were differences in FRS and ACC/AHA13 distributions by sex and age group with spread being markedly higher for younger individuals (**Figure S15**). The GRS distributions did not vary by sex or age in FINRISK.

Since the CHD outcomes in the FINRISK cohorts have been previously analyzed using Cox regression models adjusting for individual risk factors (cholesterol, blood pressure, etc.) rather than composite risk scores with predefined weights as we do here (FRS and ACC/AHA13), we sought to confirm consistent results of the n=12,676 subset used here with those prior results^8^. We fit a Cox proportional hazards model to the individual clinical risk factors (log total and HDL cholesterol, log systolic and diastolic BP, prevalent diabetes, current smoking, lipid and BP treatment, and family history of MI, adjusted for geographic location and cohort, and stratified by sex) to our data (using CHD censored at 75y), which gives a virtually identical C-index to that reported in Ripatti et al. (here C=0.870, 95% CI 0.858–0.882).

**Positive and negative predictive values**

The FRS+GRS model also showed consistently higher positive predictive value (PPV) at any given negative predictive value (NPV) for 10-year incident CHD events (**Figure S14a**). For example, in FINRISK, at an NPV of 96% the FRS+GRS achieved a PPV of 40%, compared with a PPV of 23% for FRS alone. In FHS, PPV for FRS+GRS and ACC/AHA13+GRS models was 12–13% at the same NPV, compared with PPV of 10% for the FRS or ACC/AHA13 scores (**Figure S14a**).

**Supplementary Methods**

## FINRISK study cohorts, SNP genotyping, and quality control

A total of n=16,093 individuals from three FINRISK population cohorts (FR92, FR97, and FR02) were genotyped in two batches: batch I was genotyped on the Illumina HumanHap610 platform (n=8978) and batch II on the Illumina CoreExome genotyping array (n=6972). In each batch, genotypes were further imputed using the 1000 Genomes reference panel phase I with impute2 v2.3.0^9, 10^. To minimize the potential for artificial inflation of the risk estimates^11^, we performed quality control (QC) on samples and SNPs within each batch using PLINK 1.9^12^. For SNPs, this included removing non-autosomal SNPs, removing SNPs with imputation INFO <0.4, minor allele frequency (MAF) <1%, missingness >10%, and deviation from Hardy-Weinberg equilibrium *P* <5×10^-6^. Quality control for samples included removing samples with missingness >10% (no samples removed for either batch), removal of genotyped samples without matching phenotypes (0 and 143 for batch I and II, respectively), removal of related individuals with estimated identity-by-descent (IBD) $\hat{\pi}>0.1$ (removed n=638 and n=596 in batch I and II, resp.). We also removed batch II individuals with IBD >0.1 with batch I (removed n=699). Next, we removed any samples that were included in either the DILGOM (none removed) or COROGENE (removed n=1029 and n=0 in batch I and II, respectively) studies, as these were part of the CARDIoGRAMplusC4D stage-2 meta-analysis (see below). Finally, we removed FINRISK individuals with prevalent CVD at baseline (n=312 removed). For batch I, the final dataset consisted of n=7050, across three cohorts: FR92 (n=660), FR97 (n=4761), and FR02 (n=1629), with 7,479,901 autosomal SNPs. For batch II, the final dataset consisted of n=5626 individuals, across FR92 (n=2887) and FR02 (n=2739), with 7,166,613 autosomal SNPs (see **Figure 1** in the main text for the study workflow and **Table 1** for the characteristics of each cohort).

Due to the small number of incident CHD cases in batch II (n=92 incident CHD events before age 75y) and the overlap of cohorts between the two batches, we opted to combine the two batches into one. To remove potential batch effects within the GRS (defined below), we regressed the score on the batch indicator variable and verified that there was no remaining batch effect on the GRS (*F*-test for linear regression of the score on the batch indicator variable *P*=1, *P*=0.913, and *P*=0.887 for all individuals, men only, and women only, respectively). The final combined dataset consisted of n=12,676 individuals. Outcomes regarding incident CHD events were censored at an age of 75y, as not all cohorts and geographic regions had sufficient numbers of CHD events in individuals aged >75y.

The CARDIoGRAMplusC4D Consortium meta-analysis tested 79,128 SNPs in 63,746 CHD cases and 130,681 controls from 48 individual studies. CHD was defined as a validated history of MI or revascularization (PCI or CABG) or angiographic evidence of CAD. Additional details on CHD phenotypes, including breakdown by study, are given in Deloukas et al.^1^ and Schunkert et al.^2^ The MIGen case/control dataset (dbGaP phs000294.v1.p1)^4^ consisted of 5645 samples (2782 cases, 2863 controls) assayed on the Affymetrix 6.0 array, out of which 1019 were from the Harps study (531 cases, 488 controls). The MIGen assayed SNPs were filtered by MAF <1%, missingness >1%, and deviation from Hardy-Weinberg equilibrium *P* <5×10^-6^, and individuals were filtered by missingness >1%. The WTCCC CAD case/control dataset consisted of 4864 samples (1926 cases, 2938 controls) assayed on the Affymetrix 500K arrays. We performed QC for the WTCCC assayed SNPs as has been described previously ^3^. Both datasets were imputed to 1000 Genomes Phase 1 reference panel using impute v2.3.0. The imputed SNPs were filtered by imputation score INFO <0.4.

**Framingham study cohorts, SNP genotyping, and quality control**

A total of n=9,224 individuals were genotyped on Affymetrix 500K arrays, of which 6,854 individuals could be matched to clinical attributes. We performed quality control (removing non-autosomal SNPs and SNPs with genotyping missingness >10%, MAF <1%, HWE *P*<5×10^-6^, and removing samples with missingness >10%) and mapped the SNP positions from hg18 to hg19. We then pre-phased the data using SHAPEIT v2.r790^13^ (accounting for pedigree using the ‘duohmm’ option), and next used impute2 v2.3.2 to impute the genotyped data to the 1000 Genomes Phase I integrated haplotype reference panel (June 2014). After imputation, we removed SNPs with imputation INFO <0.4. We explored GRSs based on imputed SNPs with other thresholds of INFO (>0.6, >0.7, >0.8, >0.9), however, these resulted in slightly weaker associations with CHD.

Individuals were removed if they did not have all of the clinical variables necessary for the FRS and ACC/AHA13 scores, had prevalent CHD at baseline, were aged <30y at baseline, or were in the Third cohort (due to the maximum follow-up time of <10y in that cohort). The final Framingham analysis included n=3,406 individuals in the Original and Offspring cohorts. Type 2 diabetes at each exam was defined as either fasting glucose levels of >126 mg/dL, current diagnosis for diabetes, or current treatment for diabetes.

**ARGOS SNP genotyping and quality control**

ARGOS is an “extreme phenotype” study^5^, where young familial hypercholesterolemia (FH) individuals (mean age 42y) who had experienced CHD were compared with older FH individuals (mean age 76y) without CHD. FH status was determined based on mutations in the *LDLR* gene, and recruited through the Dutch molecular screening program ‘Stichting Opsporing Erfelijke Familiare Hypercholesterolemie’.

A total of n=466 individuals were genotyped on Illumina HumanHap550K arrays. Prior to imputation, SNPs were filtered by genotyping missingness >10%, MAF <1%, and HWE *P*<5×10^-6^, and samples by missingness >10%. After QC, 522,308 SNPs and 464 samples remained (248 CHD cases, 216 controls; 240 males, 224 females). Genotypes were imputed to the 1000 Genomes Phase 3 reference panel (October 2014) using impute2 v2.3.2. Post imputation, imputed SNPs were removed if they had INFO scores <0.4.

**Principal component analysis**

Principal component analysis of the genotypes was performed in FINRISK, FHS, and ARGOS using flashpca v1.1.2 ^14^, using LD-thinned SNPs (47,697 in FINRISK, 37,610 in FHS, 54,690 in ARGOS), after removal of genomic regions chr5:44–51.5Mb, chr6:25–33.5Mb, chr8: 8–12Mb, and chr11:45–57Mb (hg19 positions).

**CHD outcome definitions in WTCCC**

CAD in the WTCCC was defined as a validated history of either myocardial infarction or coronary revascularization (coronary artery bypass surgery or percutaneous coronary angioplasty) before their 66th birthday. Verification of the history of CAD was required either from hospital records or the primary care physician.

**CHD outcome definitions in MIGen**

For MIGen, MI defined cases of early onset MI (men <50 years old, women <60 years old). MI was diagnosed from as one of:

Autopsy evidence of fatal MI

Combination of chest pain with electrocardiographic evidence of MI

Elevation of one or more cardiac biomarkers (creatine kinase or cardiac troponin)

**CHD outcome definitions in ARGOS**

CHD in ARGOS was defined as one of

- Myocardial infarction, determined by at least two of
  - Classical symptoms (>15min)
  - Specific abnormalities on electrocardiography
  - Elevated cardiac enzymes (>2× upper limit of normal)
- Percutaneous coronary intervention or other invasive procedures
- Coronary artery bypass grafting

**CHD outcome definitions in FINRISK**

The main outcome of interest was incident CHD event before age 75y, which includes all myocardial infarction (MI) events.

Coronary heart disease (CHD) was defined as falling into any of the following categories:

- I21 or I22 (**ICD-10**) / 410 (**ICD-8/9**) as the direct or as a contributing cause of death or I20-I25 (ICD-10) /410-414 (ICD-9) as the underlying cause of death
- I21 or I22 (**ICD-10**) / 410 (**ICD-8/9**) as the main or secondary diagnosis at hospital discharge.
- Coronary bypass surgery or coronary angioplasty at hospital discharge or identified from the Finnish registry of invasive cardiac procedures.

Individuals with prevalent cardiovascular disease (CVD) at baseline were excluded from analysis. CVD was defined as one of

- CHD (including myocardial infarction, MI).
- Stroke (excluding subarachnoid hemorrhage). Stroke excluded subarachnoid hemorrhage and was defined as any of the following categories:I61, I63; not I636, I64 (**ICD-10**) / 431, 4330A, 4331A, 4339A, 4340A, 4341A, 4349A, 436 (**ICD-9-Finnish modification**) / 431 (except 43101, 43191) 433, 434, 436 (**ICD-8**) as either underlying, direct, or contributing cause of death or as the main or secondary diagnosis at hospital discharge.

## CHD outcome definitions in the Framingham Heart Study

The main outcome of interest was incident CHD event before age 75y. We used the definition of CHD as employed by the Framingham study, namely, one of

- MI recognized, with diagnostic ECG (FHS event code #1)
- MI recognized, without diagnostic ECG, with enzymes and history (#2)
- MI recognized, without diagnostic ECG, with autopsy evidence (new event) (#3)
- MI unrecognized, silent (#4)
- MI unrecognized, not silent (#5)
- Angina pectoris (AP), first episode only (#6)
- Coronary insufficiency (CI), definite by both history and ECG (#7)
- Questionable MI at exam 1 (#8)
- Acute MI by autopsy, previously coded as 1 or 2 (#9)
- Death, CHD sudden, with 1 hour (#21)
- Death, CHD 1–23 hours, non sudden (#22)
- Death, CHD 24-47 hours, non sudden (#23)
- Death, CHD, 48 hours or more, non sudden (#24)

## Genomic risk scores

We utilized results from the recent worldwide CARDIoGRAMplusC4D meta-analysis (obtained from http://www.cardiogramplusc4d.org)^1^, the largest effort of its kind to date (64,000 cases and 131,000 controls), representing the best estimates so far of the effect sizes of loci associated with CHD, and increasing the total number of CHD risk loci to 46, which together have previously been estimated to explain 10.6% of CHD heritability.

Of the 79,128 Metabochip and genome-wide SNPs included in the CARDIoGRAMplusC4D meta-analysis stage-2, 69,044, 78,058, 72,727, 78,259, and 74,135 SNPs mapped to the FINRISK, Framingham, MIGen, WTCCC, and ARGOS 1000 Genomes-imputed datasets, respectively.

We used LD-thinning (PLINK 1.9 --indep-pairwise) separately on the WTCCC and MIGen datasets, over a range of *r*^2^ thresholds. For each threshold, we constructed a GRS based on the SNPs retained, together with their original weights (log odds) from the CARDIoGRAMplusC4D stage-2 meta-analysis; the final score for each person is the sum over all SNPs of each SNP’s log odds multiplied by the SNP’s minor allele dosage {0, 1, 2}. The CARDIoGRAMplusC4D meta-analysis stage-1 results were not utilized because WTCCC-CAD and MIGen were included in the stage-1 meta-analysis, and the optimization of LD-thinning for the GRS's here showed clear signs of model overfitting (maximum AUC's 0.82 and 0.73 for WTCCC-CAD and MIGen, respectively).

We also used two published genetic risk scores comprising: a 28 SNP score^6^, denoted here as *Tikkanen*, and a 27 SNP score^7^, denoted here as *Mega*. As with our GRS, the predicted score for each person was the sum of the each SNP weight multiplied by the allele dosage.

## Clinical risk scores

We examined two clinical risk scores: (i) the published Framingham Risk Score (FRS) for risk of hard CHD (myocardial infarction or coronary death) within 10 years based on the recommendations of the Adult Treatment Panel III ^15^, and (ii) the American College of Cardiologists / American Heart Association 2013 risk score (ACC/AHA13) for atherosclerotic cardiovascular disease^16^.

Baseline clinical variables included in the FRS and ACC/AHA013 risk score were: sex (male/female), blood pressure treatment (yes/no), current smoking (yes/no), type 2 diabetes status (yes/no, for ACC/AHA13 only), log total cholesterol (mg/dL), log HDL cholesterol (mg/dL), log systolic blood pressure (mm Hg), log baseline age (years), and several non-linear terms of these variables (**Tables S1 and S2**). In FINRISK, only systolic blood pressure had any missing values (n=7, 0.05% of samples); hence we used complete-case analysis. In FHS, for each person, we selected the first exam for which all baseline clinical variables were available (for Original cohort: exams 9, 10, 15, 20, and 22; for Offspring cohort: exams 1, 3, 4, 5, and 6). For computing the clinical risk scores, we converted total cholesterol and HDL cholesterol in mmol/L to mg/dL by multiplying by a factor of 38.67 ^17^.

**Statistical analysis of incident CHD**

Statistical analyses were conducted in the R statistical environment v3.1.3 and in Stata 13.1.

In the FINRISK analysis, following complementary log-log plots of the Kaplan-Meier survival curves (**Figure S16**) and the scaled Schoenfeld residual test (cox.zph in R package survival, global *P*=0.079), we established approximate proportionality of the hazards for different geographic locations and cohorts within each sex. We used sex-stratified Cox proportional hazard models, with age as the time scale, adjusting for geographic location and cohort (FR92, FR97, FR02), using the R package survival^18^, to model the contribution of the GRS and the clinical risk scores to CHD risk. Within each model we standardized each predictor (clinical risk score and GRS) to zero-mean and unit variance.

Competing risk models were fit using cause-specific Cox regression (in Stata), using age as the time scale, and their cumulative incidence curves were computed^19, 20^. For the competing risk model, we coded the outcome for each individual to be one of: incident CHD, non-CHD death, or censored. Analysis of possible time-varying effects was conducted in Stata using stcox with the option tvc. For empirical competing risk cumulative incidence curve, we used survfit in R with multi-state counting process formulation (Aalen-Johansen estimator, **Figure S10**)

Discrimination of time to event was assessed using Harrell’s C-index^21^ and its standard error, and difference in C-index between two models was assessed using the correlated C-index jackknife test^22^. We assessed the calibration of the models for incident CHD <10y (predicted absolute risk versus empirical risk) using the Nam-D’Agostino method of comparing *m*=5 quintiles of predicted risk with the empirical Kaplan-Meier risk^23, 24^, compared against the χ^2^_m-1_ distribution. The Tikkanen score and GRS showed minor deviation from calibration (*P*=0.034 and *P*=0.037, respectively), however, all other models combining the GRS with clinical scores had good calibration (maximum χ^2^_4df_<7, **Figure S17**).

Based on the predicted absolute risks of CHD within 10y and the observed binary outcomes of CHD within 10y, we evaluated the continuous Net Reclassification Index (NRI), the Categorical Net Reclassification Index (risk cutoffs of 0–7.5%, 7.5–10%, 10–20%, and 20–100%), and Integrated Discrimination Index (IDI)^25, 26^, using the R package Hmisc^21^.

**Meta analyses**

Fixed-effect inverse-variance weighted meta-analyses were conducted using the metagen function in the R package meta (v4.4-1). Heterogeneity was assessed using Cochran’s *Q*-statistic ^27^ and the *I*^2^-statistic^28, 29^. The heterogeneity p-value reported here is for the *Q-*statistic, evaluated using the χ^2^ distribution with df=*k*-1 degrees of freedom, where *k* is the number of studies in the meta-analysis. The *I*^2^ statistic is calculated as *I*^2^ = 100×(*Q* – df) / *Q*, and thus quantifies the percentage of total variation across the meta-analysed studies that is due to heterogeneity^29^, unlike Cochran’s *Q*-test that does not summarise the magnitude of heterogeneity but only whether it deviates significantly from the null.

**References**

1. CARDIoGRAMplusC4D Consortium, Deloukas P, Kanoni S, Willenborg C, Farrall M, Assimes TL, Thompson JR, Ingelsson E, Saleheen D, Erdmann J, Goldstein BA, Stirrups K, Konig IR, Cazier JB, Johansson A, Hall AS, Lee JY, Willer CJ, Chambers JC, Esko T, Folkersen L, Goel A, Grundberg E, Havulinna AS, Ho WK, Hopewell JC, Eriksson N, Kleber ME, Kristiansson K, Lundmark P, Lyytikainen LP, Rafelt S, Shungin D, Strawbridge RJ, Thorleifsson G, Tikkanen E, Van Zuydam N, Voight BF, Waite LL, Zhang W, Ziegler A, Absher D, Altshuler D, Balmforth AJ, Barroso I, Braund PS, Burgdorf C, Claudi-Boehm S, Cox D, Dimitriou M, Do R, DIAGRAM Consortium, CARDIOGENICS Consortium, Doney AS, El Mokhtari N, Eriksson P, Fischer K, Fontanillas P, Franco-Cereceda A, Gigante B, Groop L, Gustafsson S, Hager J, Hallmans G, Han BG, Hunt SE, Kang HM, Illig T, Kessler T, Knowles JW, Kolovou G, Kuusisto J, Langenberg C, Langford C, Leander K, Lokki ML, Lundmark A, McCarthy MI, Meisinger C, Melander O, Mihailov E, Maouche S, Morris AD, Muller-Nurasyid M, MuTHeR Consortium, Nikus K, Peden JF, Rayner NW, Rasheed A, Rosinger S, Rubin D, Rumpf MP, Schafer A, Sivananthan M, Song C, Stewart AF, Tan ST, Thorgeirsson G, van der Schoot CE, Wagner PJ, Wellcome Trust Case Control Consortium, Wells GA, Wild PS, Yang TP, Amouyel P, Arveiler D, Basart H, Boehnke M, Boerwinkle E, Brambilla P, Cambien F, Cupples AL, de Faire U, Dehghan A, Diemert P, Epstein SE, Evans A, Ferrario MM, Ferrieres J, Gauguier D, Go AS, Goodall AH, Gudnason V, Hazen SL, Holm H, Iribarren C, Jang Y, Kahonen M, Kee F, Kim HS, Klopp N, Koenig W, Kratzer W, Kuulasmaa K, Laakso M, Laaksonen R, Lee JY, Lind L, Ouwehand WH, Parish S, Park JE, Pedersen NL, Peters A, Quertermous T, Rader DJ, Salomaa V, Schadt E, Shah SH, Sinisalo J, Stark K, Stefansson K, Tregouet DA, Virtamo J, Wallentin L, Wareham N, Zimmermann ME, Nieminen MS, Hengstenberg C, Sandhu MS, Pastinen T, Syvanen AC, Hovingh GK, Dedoussis G, Franks PW, Lehtimaki T, Metspalu A, Zalloua PA, Siegbahn A, Schreiber S, Ripatti S, Blankenberg SS, Perola M, Clarke R, Boehm BO, O'Donnell C, Reilly MP, Marz W, Collins R, Kathiresan S, Hamsten A, Kooner JS, Thorsteinsdottir U, Danesh J, Palmer CN, Roberts R, Watkins H, Schunkert H, Samani NJ. Large-scale association analysis identifies new risk loci for coronary artery disease. Nat Genet 2013;**45**(1):25-33.

2. Schunkert H, Konig IR, Kathiresan S, Reilly MP, Assimes TL, Holm H, Preuss M, Stewart AF, Barbalic M, Gieger C, Absher D, Aherrahrou Z, Allayee H, Altshuler D, Anand SS, Andersen K, Anderson JL, Ardissino D, Ball SG, Balmforth AJ, Barnes TA, Becker DM, Becker LC, Berger K, Bis JC, Boekholdt SM, Boerwinkle E, Braund PS, Brown MJ, Burnett MS, Buysschaert I, Cardiogenics, Carlquist JF, Chen L, Cichon S, Codd V, Davies RW, Dedoussis G, Dehghan A, Demissie S, Devaney JM, Diemert P, Do R, Doering A, Eifert S, Mokhtari NE, Ellis SG, Elosua R, Engert JC, Epstein SE, de Faire U, Fischer M, Folsom AR, Freyer J, Gigante B, Girelli D, Gretarsdottir S, Gudnason V, Gulcher JR, Halperin E, Hammond N, Hazen SL, Hofman A, Horne BD, Illig T, Iribarren C, Jones GT, Jukema JW, Kaiser MA, Kaplan LM, Kastelein JJ, Khaw KT, Knowles JW, Kolovou G, Kong A, Laaksonen R, Lambrechts D, Leander K, Lettre G, Li M, Lieb W, Loley C, Lotery AJ, Mannucci PM, Maouche S, Martinelli N, McKeown PP, Meisinger C, Meitinger T, Melander O, Merlini PA, Mooser V, Morgan T, Muhleisen TW, Muhlestein JB, Munzel T, Musunuru K, Nahrstaedt J, Nelson CP, Nothen MM, Olivieri O, Patel RS, Patterson CC, Peters A, Peyvandi F, Qu L, Quyyumi AA, Rader DJ, Rallidis LS, Rice C, Rosendaal FR, Rubin D, Salomaa V, Sampietro ML, Sandhu MS, Schadt E, Schafer A, Schillert A, Schreiber S, Schrezenmeir J, Schwartz SM, Siscovick DS, Sivananthan M, Sivapalaratnam S, Smith A, Smith TB, Snoep JD, Soranzo N, Spertus JA, Stark K, Stirrups K, Stoll M, Tang WH, Tennstedt S, Thorgeirsson G, Thorleifsson G, Tomaszewski M, Uitterlinden AG, van Rij AM, Voight BF, Wareham NJ, Wells GA, Wichmann HE, Wild PS, Willenborg C, Witteman JC, Wright BJ, Ye S, Zeller T, Ziegler A, Cambien F, Goodall AH, Cupples LA, Quertermous T, Marz W, Hengstenberg C, Blankenberg S, Ouwehand WH, Hall AS, Deloukas P, Thompson JR, Stefansson K, Roberts R, Thorsteinsdottir U, O'Donnell CJ, McPherson R, Erdmann J, Consortium CA, Samani NJ. Large-scale association analysis identifies 13 new susceptibility loci for coronary artery disease. Nat Genet 2011;**43**(4):333-8.

3. The Wellcome Trust Case Control Consortium. Genome-wide association study of 14,000 cases of seven common diseases and 3,000 shared controls. Nature 2007;**447**:661-78.

4. Myocardial Infarction Genetics Consortium, Kathiresan S, Voight BF, Purcell S, Musunuru K, Ardissino D, Mannucci PM, Anand S, Engert JC, Samani NJ, Schunkert H, Erdmann J, Reilly MP, Rader DJ, Morgan T, Spertus JA, Stoll M, Girelli D, McKeown PP, Patterson CC, Siscovick DS, O'Donnell CJ, Elosua R, Peltonen L, Salomaa V, Schwartz SM, Melander O, Altshuler D, Ardissino D, Merlini PA, Berzuini C, Bernardinelli L, Peyvandi F, Tubaro M, Celli P, Ferrario M, Fetiveau R, Marziliano N, Casari G, Galli M, Ribichini F, Rossi M, Bernardi F, Zonzin P, Piazza A, Mannucci PM, Schwartz SM, Siscovick DS, Yee J, Friedlander Y, Elosua R, Marrugat J, Lucas G, Subirana I, Sala J, Ramos R, Kathiresan S, Meigs JB, Williams G, Nathan DM, MacRae CA, O'Donnell CJ, Salomaa V, Havulinna AS, Peltonen L, Melander O, Berglund G, Voight BF, Kathiresan S, Hirschhorn JN, Asselta R, Duga S, Spreafico M, Musunuru K, Daly MJ, Purcell S, Voight BF, Purcell S, Nemesh J, Korn JM, McCarroll SA, Schwartz SM, Yee J, Kathiresan S, Lucas G, Subirana I, Elosua R, Surti A, Guiducci C, Gianniny L, Mirel D, Parkin M, Burtt N, Gabriel SB, Samani NJ, Thompson JR, Braund PS, Wright BJ, Balmforth AJ, Ball SG, Hall A, Wellcome Trust Case Control C, Schunkert H, Erdmann J, Linsel-Nitschke P, Lieb W, Ziegler A, Konig I, Hengstenberg C, Fischer M, Stark K, Grosshennig A, Preuss M, Wichmann HE, Schreiber S, Schunkert H, Samani NJ, Erdmann J, Ouwehand W, Hengstenberg C, Deloukas P, Scholz M, Cambien F, Reilly MP, Li M, Chen Z, Wilensky R, Matthai W, Qasim A, Hakonarson HH, Devaney J, Burnett MS, Pichard AD, Kent KM, Satler L, Lindsay JM, Waksman R, Knouff CW, Waterworth DM, Walker MC, Mooser V, Epstein SE, Rader DJ, Scheffold T, Berger K, Stoll M, Huge A, Girelli D, Martinelli N, Olivieri O, Corrocher R, Morgan T, Spertus JA, McKeown P, Patterson CC, Schunkert H, Erdmann E, Linsel-Nitschke P, Lieb W, Ziegler A, Konig IR, Hengstenberg C, Fischer M, Stark K, Grosshennig A, Preuss M, Wichmann HE, Schreiber S, Holm H, Thorleifsson G, Thorsteinsdottir U, Stefansson K, Engert JC, Do R, Xie C, Anand S, Kathiresan S, Ardissino D, Mannucci PM, Siscovick D, O'Donnell CJ, Samani NJ, Melander O, Elosua R, Peltonen L, Salomaa V, Schwartz SM, Altshuler D. Genome-wide association of early-onset myocardial infarction with single nucleotide polymorphisms and copy number variants. Nat Genet 2009;**41**(3):334-41.

5. Versmissen J, Oosterveer DM, Yazdanpanah M, Dehghan A, Holm H, Erdman J, Aulchenko YS, Thorleifsson G, Schunkert H, Huijgen R, Vongpromek R, Uitterlinden AG, Defesche JC, van Duijn CM, Mulder M, Dadd T, Karlsson HD, Ordovas J, Kindt I, Jarman A, Hofman A, van Vark-van der Zee L, Blommesteijn-Touw AC, Kwekkeboom J, Liem AH, van der Ouderaa FJ, Calandra S, Bertolini S, Averna M, Langslet G, Ose L, Ros E, Almagro F, de Leeuw PW, Civeira F, Masana L, Pinto X, Simoons ML, Schinkel AF, Green MR, Zwinderman AH, Johnson KJ, Schaefer A, Neil A, Witteman JC, Humphries SE, Kastelein JJ, Sijbrands EJ. Identifying genetic risk variants for coronary heart disease in familial hypercholesterolemia: an extreme genetics approach. Eur J Hum Genet 2015;**23**(3):381-7.

6. Tikkanen E, Havulinna AS, Palotie A, Salomaa V, Ripatti S. Genetic risk prediction and a 2-stage risk screening strategy for coronary heart disease. Arterioscler Thromb Vasc Biol 2013;**33**(9):2261-6.

7. Mega JL, Stitziel NO, Smith JG, Chasman DI, Caulfield MJ, Devlin JJ, Nordio F, Hyde CL, Cannon CP, Sacks FM, Poulter NR, Sever PS, Ridker PM, Braunwald E, Melander O, Kathiresan S, Sabatine MS. Genetic risk, coronary heart disease events, and the clinical benefit of statin therapy: an analysis of primary and secondary prevention trials. Lancet 2015;**385**(9984):2264-71.

8. Ripatti S, Tikkanen E, Orho-Melander M, Havulinna AS, Silander K, Sharma A, Guiducci C, Perola M, Jula A, Sinisalo J, Lokki ML, Nieminen MS, Melander O, Salomaa V, Peltonen L, Kathiresan S. A multilocus genetic risk score for coronary heart disease: case-control and prospective cohort analyses. Lancet 2010;**376**(9750):1393-400.

9. Howie B, Fuchsberger C, Stephens M, Marchini J, Abecasis GR. Fast and accurate genotype imputation in genome-wide association studies through pre-phasing. Nat Genet 2012;**44**(8):955-9.

10. Howie BN, Donnelly P, Marchini J. A flexible and accurate genotype imputation method for the next generation of genome-wide association studies. PLoS Genet 2009;**5**(6):e1000529.

11. Wray NR, Yang J, Hayes BJ, Price AL, Goddard ME, Visscher PM. Pitfalls of predicting complex traits from SNPs. Nat Rev Genet 2013;**14**(7):507-15.

12. Chang CC, Chow CC, Tellier LC, Vattikuti S, Purcell SM, Lee JJ. Second-generation PLINK: rising to the challenge of larger and richer datasets. Gigascience 2015;**4**:7.

13. Delaneau O, Marchini J, Zagury JF. A linear complexity phasing method for thousands of genomes. Nat Methods 2012;**9**(2):179-81.

14. Abraham G, Inouye M. Fast principal component analysis of large-scale genome-wide data. PLoS One 2014;**9**(4):e93766.

15. Expert Panel on Detection, Evaluation, And Treatment of High Blood Cholesterol in Adults. Executive Summary of The Third Report of The National Cholesterol Education Program (NCEP) Expert Panel on Detection, Evaluation, And Treatment of High Blood Cholesterol In Adults (Adult Treatment Panel III). JAMA 2001;**285**(19):2486-97.

16. Goff DC, Jr., Lloyd-Jones DM, Bennett G, Coady S, D'Agostino RB, Gibbons R, Greenland P, Lackland DT, Levy D, O'Donnell CJ, Robinson JG, Schwartz JS, Shero ST, Smith SC, Jr., Sorlie P, Stone NJ, Wilson PW, Jordan HS, Nevo L, Wnek J, Anderson JL, Halperin JL, Albert NM, Bozkurt B, Brindis RG, Curtis LH, DeMets D, Hochman JS, Kovacs RJ, Ohman EM, Pressler SJ, Sellke FW, Shen WK, Smith SC, Jr., Tomaselli GF. 2013 ACC/AHA guideline on the assessment of cardiovascular risk: a report of the American College of Cardiology/American Heart Association Task Force on Practice Guidelines. Circulation 2014;**129**(25 Suppl 2):S49-73.

17. Rugge B, Balshem H, Sehgal R, Relevo R, Gorman P, Helfand M. In. *Screening and Treatment of Subclinical Hypothyroidism or Hyperthyroidism*. Rockville (MD); 2011.

18. Therneau TM, Grambsch PM. *Modeling survival data : extending the Cox model*. New York: Springer; 2000.

19. Wolbers M, Blanche P, Koller MT, Witteman JC, Gerds TA. Concordance for prognostic models with competing risks. Biostatistics 2014;**15**(3):526-39.

20. Wolbers M, Koller MT, Witteman JC, Steyerberg EW. Prognostic models with competing risks: methods and application to coronary risk prediction. Epidemiology 2009;**20**(4):555-61.

21. Harrell FE. *Regression modeling strategies : with applications to linear models, logistic regression, and survival analysis*. New York: Springer; 2001.

22. Antolini L, Nam B-H, D'Agostino RB. Inference on Correlated Discrimination Measures in Survival Analysis: A Nonparametric Approach. Communications in Statistics - Theory and Methods 2004;**33**(9):2117-2135.

23. D'Agostino RB, Nam B-H. Evaluation of the Performance of Survival Analysis Models: Discrimination and Calibration Measures. In: Rao CR, Balakrishnan N, (eds). *Advances in survival analysis*: Elsevier North-Holland; 2004, 1-25.

24. D'Agostino RB, Sr., Pencina MJ, Massaro JM, Coady S. Cardiovascular Disease Risk Assessment: Insights from Framingham. Glob Heart 2013;**8**(1):11-23.

25. Pencina MJ, D'Agostino RB, Sr., Steyerberg EW. Extensions of net reclassification improvement calculations to measure usefulness of new biomarkers. Stat Med 2011;**30**(1):11-21.

26. Pencina MJ, D'Agostino RB, Sr., D'Agostino RB, Jr., Vasan RS. Evaluating the added predictive ability of a new marker: from area under the ROC curve to reclassification and beyond. Stat Med 2008;**27**(2):157-72; discussion 207-12.

27. Cochran WG. The Comparison of Percentages in Matched Samples. Biometrika 1950;**37**(3-4):256-266.

28. Higgins JP, Thompson SG. Quantifying heterogeneity in a meta-analysis. Stat Med 2002;**21**(11):1539-58.

29. Higgins JP, Thompson SG, Deeks JJ, Altman DG. Measuring inconsistency in meta-analyses. BMJ 2003;**327**(7414):557-60.

**Supplementary Tables**

**Table S1: Framingham risk score (FRS) function for 10y to hard CHD**. Reproduced from http://www.framinghamheartstudy.org/risk-functions/coronary-heart-disease/hard-10-year-risk.php (accessed 25 Aug 2014).

| **Variable** | **Men** | **Women** |
| --- | --- | --- |
| Ln Age (y) | 52.010 | 31.764 |
| Ln Total Cholesterol (mg/dL) | 20.014 | 22.465 |
| Ln HDL Cholesterol (mg/dL) | -0.906 | -1.188 |
| Ln Systolic BP (mm Hg) | 1.306 | 2.553 |
| Treatment for hypertension (1=yes, 0=no) | 0.242 | 0.420 |
| Current Smoker (1=yes, 0=no) | 12.096 | 13.075 |
| Ln Age × Ln Total Cholesterol | -4.605† | -5.061‡ |
| Ln Age × Current Smoker | -2.844 | -2.997 |
| Ln Age × Ln Age | -2.933 | 0 |

† If Age > 70 then use Ln(70) × Current Smoker

‡ If Age > 78 then use Ln(78) × Current Smoker

A weight of 0 represents no effect.

## Table S2: ACC/AHA 2013 risk function for 10y risk of hard atherosclerotic CVD (ASCVD) individuals of European ancestry (“White”)

| **Variable** | **Men** | **Women** |
| --- | --- | --- |
| Ln Age (y) | 12.344 | -29.799 |
| Ln Age, squared | 0 | 4.884 |
| Ln Total Cholesterol (mg/dL) | 11.853 | 13.540 |
| Ln Age × Ln Total Cholesterol | -2.664 | -3.114 |
| Ln HDL-C (mg/dL) | -7.990 | -13.578 |
| Ln Age × Ln HDL-C | 1.769 | 3.149 |
| Ln Treated Systolic BP (mm Hg) | 1.797 | 2.019 |
| Ln Untreated Systolic BP (mm Hg) | 1.764 | 1.957 |
| Current Smoker (1=yes, 0=no) | 7.837 | 7.574 |
| Ln Age × Current Smoker | -1.795 | -1.665 |
| Diabetes (1=yes, 0=no) | 0.658 | 0.661 |

A weight of 0 represents no effect.

We used a factor of 38.67 to convert from mmol/L units of cholesterol (total cholesterol, HDL, or LDL) to mg/dL units (http://www.ncbi.nlm.nih.gov/books/NBK83505/).

**Table S3: Improvement in 10y C-index of the 49K GRS over other genomic risk scores in FINRISK and FHS.** 95% confidence intervals and p-values are from the jackknife correlated C-index test. Meta-analysis was conducted using the inverse-variance weighted fixed-effect method.

|  | **FINRISK (batch I, n=7046)** | **FHS (n=3046)** | **Meta-analysis** |
| --- | --- | --- | --- |
| **Tikkanen** | +2.1% (1.3–2.9%)  *P* < 1×10^-6^ | +1.0% (-0.5–2.5%)  *P* = 0.18 | +1.9% (1.2–2.6%)  *P* < 1×10^-6^ |
| **Mega** | +2.3% (1.5–3.1%)  *P* < 1×10^-6^ | +1.4% (-0.1–3%)  *P* = 0.0616 | +2.1% (1.4–2.8%)  *P* < 1×10^-6^ |
| **FDR153** | +2.1% (1.3–2.9%)  *P* < 1×10^-6^ | +0.8% (-0.6–2.3%)  *P* = 0.26 | +1.8% (1.1–2.5%)  *P* < 1×10^-6^ |

* In FINRISK batch I, 4 individuals were removed due to missing clinical variables.

Tikkanen score meta-analysis heterogeneity: *I*^2^ = 0.42, *Q* = 1.72, *P* = 0.189; Mega score meta-analysis heterogeneity: *I*^2^ = 0.007, *Q* = 1.01, *P* = 0.316; FDR153 meta-analysis heterogeneity: *I*^2^ = 0.574, *Q* = 2.35, *P* = 0.125.

**Table S4: C-index for 10y incident CHD (95% confidence interval), using risk models in FHS and FINRISK.** The C-index was computed based on the absolute cumulative risk of CHD up to 10y from baseline. The reference model for FHS includes the cohort and is stratified by sex; for FINRISK it includes the cohort, location (east/west), and is stratified by sex.

| **Study** | **Reference** | **FRS** | **ACC/AHA13** | **FRS+GRS** | **ACC/AHA13+GRS** |
| --- | --- | --- | --- | --- | --- |
| FHS | 0.684  (0.649–0.719) | 0.731  (0.698–0.765) | 0.729  (0.695–0.762) | 0.742  (0.709–0.775) | 0.739  (0.706–0.773) |
| FINRISK | 0.833  (0.820–0.846) | 0.848  (0.836–0.860) | 0.851  (0.839–0.863) | 0.866  (0.854–0.877) | 0.867  (0.855–0.879) |

**Supplementary Figures**

**Figure S1: Association of the genomic risk scores with CHD (case/control).** Comparing the approach of constructing GRS by LD-thinning all mapped CARDIoGRAMplusC4D SNPs (72,000–78,000) with the GRS using the 153 SNPs selected at false discovery rate (FDR) <5% and *r*^2^ <0.2 by CARDIoGRAMplusC4D. Results are given within the WTCCC-CAD (*n*=4864), MIGen-Harps (*n*=1019), and in fixed-effect meta-analysis of WTCCC-CAD and MIGen-Harps datasets. Panels (a) and (c): the odds ratio (95% CI) and AUC (95% CI) for the 153 SNPs; Panels (b) and (d): the odds ratio (95% CI) and AUC (95% CI) for LD thinning the SNPs as a function of the *r*^2^ threshold used. Panel (e): The number of SNPs selected though LD-thinning within each dataset.

**Figure S2: Density plots of the 49K GRS in individuals with CHD before age 75y, in FHS and FINRISK.** The GRS was standardised to zero mean and unit variance within each dataset.

**Figure S3:** **Association of the genomic risk score with time to incident CHD (hazard ratios and 95% CIs) in FINRISK.** Based on sex-stratified Cox PH models in the training data, for (1) GRS only (unadjusted), (2) GRS adjusting for FRS score, location, and cohort, (3) GRS adjusting for ACC/AHA13 score, location, and cohort, (4) GRS adjusting for lipid treatment at baseline (Y/N), location, and cohort, (5) GRS adjusting for MI family history, (6) GRS adjusting for location, cohort, prevalent type-2 diabetes at baseline (Y/N), log total cholesterol, log HDL, log systolic BP, smoking status (Y/N), lipid treatment (Y/N), and MI family history (Y/N), (7) GRS adjusting for location, cohort, and top 5 principal components (PCs) of the genotypes (the PCs explained 11% of the variation in the GRS).

**Figure S4:** **Association of the genomic risk score with time to incident CHD (hazard ratios and 95% CIs) in FHS.** Based on sex-stratified Cox PH models in the training data, for (1) GRS only (adjusted for cohort), (2) GRS adjusting for FRS score and cohort, (3) GRS adjusting for ACC/AHA13 score and cohort, (4) GRS adjusting for cohort, prevalent type-2 diabetes at baseline (Y/N), log total cholesterol, log HDL, log systolic BP, and smoking status (Y/N), and blood pressure treatment (Y/N) (5) GRS adjusting for cohort and prevalent type-2 diabetes at baseline, and (6) GRS adjusting for cohort and 5 principal components (PCs) of the genotypes (the PCs explained 2% of the variation in the GRS).

**Figure S5: The GRS is largely independent of clinical risk scores in (a) FINRISK and (b) FHS.** The diagonal panels show the marginal density plots of each variable (standardized to zero-mean and unit-variance). Squared Pearson correlations and p-values are from linear regression.

**
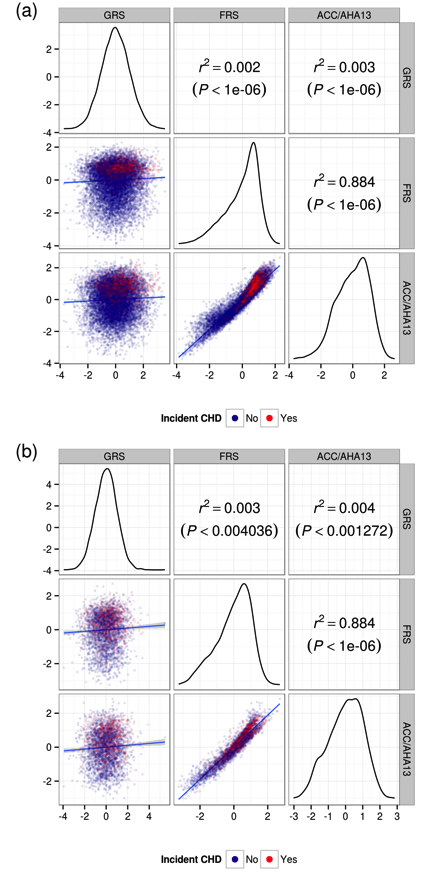
**

**Figure S6**: **Fixed-effect inverse-variance weighted** **meta-analysis of difference in C-index (95% CI) for time to incident CHD event within 10y relative to the reference model in the FINRISK and FHS cohorts**, for (a) FRS+GRS, individuals <60y at baseline, (b) FRS+GRS, individuals ≥60y at baseline, (c) ACC/AHA13+GRS, individuals <60y at baseline, and (d) ACC/AHA13+GRS, individuals ≥60y at baseline. Based on Cox proportional hazard models fit to all individuals, within each study (FINRISK and FHS).

**Figure S7: Meta-analysis of the categorical Net Reclassification Improvement (NRI) from adding the GRS to the (a) FRS and (b) ACC/AHA13 score, based on absolute risk categories of CHD <10y of 0-7.5%, 7.5-10%, 10-20%, and 20-100%.**

**Figure S8: Meta-analysis of the continuous Net Reclassification Improvement (NRI) from adding the GRS to the (a) FRS and (b) ACC/AHA13 score, based on absolute risk of CHD <10y.**

**Figure S9: Meta-analysis of the continuous Integrated Discrimination Improvement (IDI) from adding the GRS to the (a) FRS and (b) ACC/AHA13 score, based on absolute risk of CHD <10y.**

**Figure S10: Cumulative risk and incidence curves from a competing risk analysis in FINRISK.** The Cox model of the GRS was fitted to all data (n=12,676) using age as the time scale and stratifying by sex. The competing risk cumulative incidence curve was computed accounting for 3 possible outcomes: incident CHD, non-CHD death, and censoring.

**Figure S11: Kaplan-Meier curves for incident CHD event risk stratified by GRS quintiles and smoking status at baseline, for men and women in the FHS cohorts.**

**Figure S12: Kaplan-Meier curves for incident CHD risk stratified by GRS quintiles and tertiles of systolic blood pressure at baseline, for men and women in (a) FINRISK and (b) FHS.** For FINRISK, low SBP: <125 mmHg; medium SBP: 125–141 mmHg; high SBP: >141 mmHg. For FHS, low SBP: <116 mmHg; medium SBP: 116–130 mmHg; high SBP: >130 mmHg. Dotted lines indicate 95% CI's.

**Figure S13: Kaplan-Meier curves for incident CHD risk stratified by GRS quintiles and tertiles of total cholesterol (total C) at baseline, for men and women in (a) FINRISK and (b) FHS**. For FINRISK, low total C: <195 mg/dL; medium total C: 195–230 mg/dL; high total C: >230 mg/dL. For FHS, low total C: <190 mg/dL; medium total C: 190–226 mg/dL, high total C: >226 mg/dL. Dotted lines indicate 95% CIs.

**Figure S14: (a) Positive predictive value (PPV) vs. negative predictive value (NPV) and (b) ROC curves, for incident CHD event within 10 years in FINRISK and FHS, within age groups at baseline.** Using boostrap (*B*=200) LOESS-smoothed estimates. The empirical prevalence of incident CHD events within 10 years was 4.1% in FINRISK and 4.4% in FHS. The reference Cox model included: (i) for FINRISK: geographic location and cohort, stratified by sex, (ii) for FHS: cohort, stratified by sex.

**Figure S15: Density plots of the FRS, ACC/AHA13, and GRS scores by sex and age category (<60y and** ≥**60y) in FINRISK.** The scores were standardized to mean of zero and standard deviation of one over all individuals.

**Figure S16:** **Kaplan-Meier curves of survival (on complementary log-log scale) stratified by (a) cohort (FR92, FR97, FR02) and sex and (b) geographic location and sex, in FINRISK.**

**Figure S17**: **Calibration of absolute risk of CHD <10y for the various genomic risk scores and their combinations with the clinical risk scores, in the FINRISK and FRS cohorts**. In each panel, the predicted risk versus Kaplan-Meier observed risk was compared in m=5 quintiles using the Nam-D’Agostino χ^2^_4df_ calibration statistic**.**
