# Supplementary material for: Genomic prediction of coronary heart disease
Source: Eur Heart J. 2016 Sep 21;37(43):3267–78. doi: 10.1093/eurheartj/ehw450 (PMC5146693; doi:10.1093/eurheartj/ehw450)
Supplement: Supplementary Data [file supp_ehw450_ehw450.DC1.html]

Supplementary Data | European Heart Journal

## Supplementary Data

files

- Supplementary Data - docx file
